# Quality Assessment of Prune Jam with Different Concentration Methods Based on Physicochemical Properties, GC-IMS, and Intelligent Sensory Analysis

**DOI:** 10.3390/foods14122084

**Published:** 2025-06-13

**Authors:** Rui Yang, Langhan Zhao, Wei Wang, Qingping Du, Wei Li, Tongle Sun, Shihao Huang

**Affiliations:** College of Food Science and Pharmacy, Xinjiang Agricultural University, Urumqi 830052, China; yrui1220@163.com (R.Y.); 19143702998@163.com (L.Z.);

**Keywords:** prune jam, E-tongue, E-nose, GC-IMS, sensory evaluation

## Abstract

This study systematically investigated the impacts of four concentration methods—vacuum freezing concentration (VFC), microwave vacuum concentration (MVC), atmospheric thermal concentration (ATC), and vacuum thermal concentration (VTC)—on the quality and volatile compounds of prune jam. Advanced analytical techniques, including electronic tongue, electronic nose, gas chromatography–ion mobility spectrometry (GC-IMS), and multivariate statistical methods (principal component analysis, partial least squares discriminant analysis), were employed to evaluate physicochemical properties and flavor profiles. Results showed that non-thermal methods (particularly VFC) significantly outperformed thermal methods (ATC/VTC) in nutrient preservation. For instance, VFC retained 91.4% of ascorbic acid and limited dietary fiber loss to 4.55%, while ATC caused up to 60.1% ascorbic acid degradation and 51.75% dietary fiber loss. In terms of color stability, VFC induced a 1.04-fold increase in browning index (BI) and a 2.54-fold increase in total color difference (ΔE), significantly lower than ATC’s 1.6-fold BI increase and 7.26-fold ΔE rise. GC-IMS identified 42 volatile compounds, categorized into aldehydes (17), alcohols (9), esters (7), etc. Multivariate analysis screened 15 key flavor compounds (VIP > 1, *p* < 0.05), such as ethyl acetate and methanol, revealing that non-thermal methods better preserved the characteristic sweet–sour flavor and reduced off-flavor formation. These findings highlight VFC’s superiority in maintaining nutritional and sensory quality, providing scientific guidance for industrial jam production and flavor optimization in fruit processing.

## 1. Introduction

Prunes (*Prunus domestica* L.), which belong to the genus Prunus in the Rosaceae family [1], are low-calorie, low-fat, and low-sugar fruits with remarkable nutritional value. Rich in bioactive compounds, such as dietary fiber and polyphenols [2], prunes exhibit various physiological functions, including antioxidant activity, immune enhancement, and cardiovascular protection [3]. However, the prune has a short ripening season and exhibits high post-harvest respiratory activity, thereby causing rapid deterioration of fresh fruits [4]. Processing prunes into products such as dried prunes, preserved prunes, and prune jam can significantly improve their commercial viability and economic benefits [5,6]. Prune jam, a major processed product, is produced through sequential technological steps including washing, juicing, and concentration. The final gel-like product, formulated with sugar, acidulants, and thickening agents, is favored by consumers for its low-calorie content, high dietary fiber content, and distinctive flavor profile [7]. Concentration, a critical step in jam production, directly influences product quality. However, the differential effects of various concentration methods on the physicochemical and sensory properties of prune jam remain to be investigated.

Although concentration is crucial for the stability of jams, there remains a lack of systematic comparison between thermal concentration methods (ATC, VTC) and non-thermal approaches (VFC, MVC) regarding their impacts on nutritional composition and flavor integrity in prune jam. Thermal methods ATC and VTC induce significant degradation of heat-sensitive nutrients such as ascorbic acid and polyphenols through prolonged high-temperature processing, while generating undesirable off-flavors [7]. In contrast, non-thermal concentration techniques (VFC and MVC) mitigate these losses by reducing thermal stress, offering novel pathways for prune jam processing. However, their application in prune jam production remains insufficiently explored, with existing studies highlighting critical research gaps in operational optimization and quality evaluation [8,9]. Fadavi et al. [10] demonstrated that vacuum evaporation significantly increases lycopene retention in tomato juice concentrate. Freeze concentration has been shown to improve the stability of fish protein hydrolysates while simultaneously preserving their nutritional and bioactive properties [7]. VFC has been proven superior in maintaining both quality characteristics and aromatic profiles of sea buckthorn clarified juice [11]. Comparative studies reveal that Dangshan pear paste produced through vacuum evaporation contains significantly higher levels of total phenols, ascorbic acid, and total acids, and a lower browning index, than conventional atmospheric concentration methods [12]. Non-thermal concentration techniques, compared with thermal approaches, exhibit marked advantages in minimizing volatile compound loss, mitigating color browning, retaining bioactive constituents, and enhancing commercial viability [13]. Traditional sensory evaluation and single-technique analytical methods have been proven inadequate for capturing the dynamic complexity of flavor evolution. The hypothesis of this study is that non-thermal concentration better preserves bioactive compounds (e.g., polyphenols, ascorbic acid) and characteristic fruit aromas (e.g., ethyl acetate) when compared with thermal methods, a proposition that has been systematically validated through multimodal analytical instrumentation.

To address this challenge, an integrated analytical framework incorporating an E-tongue (objective taste quantification), an E-nose (volatile aroma profiling), and GC-IMS (trace compound detection), and integrating multivariate statistical methodologies—PCA and partial least squares discriminant analysis (PLS-DA).

This study investigates the effects of four concentration methods—ATC, VTC, MVC, and VFC—on the physicochemical and sensory quality parameters of prune jam. Analyzed indices include color parameters (CPs), browning index (BI), pH, titratable acidity (TA), and ascorbic acid (AA). Advanced analytical technologies, namely an E-tongue (for non-volatile taste profiling), an E-nose (for volatile aroma characterization), and GC-IMS (for trace compound identification), were systematically employed to characterize both volatile and non-volatile flavor compounds and construct comprehensive flavor fingerprint profiles. A comparative analysis of quality attributes and flavor profiles across different concentration methods was conducted to identify the optimal processing method. The findings aim to provide scientific guidance for optimizing prune jam processing technology, enhancing product quality consistency, and promoting the industrialization of prune-derived food products, thereby offering actionable insights for the fruit processing industry.

## 2. Materials and Methods

### 2.1. Experimental Materials and Equipment

#### 2.1.1. Materials

In October 2024, European prune fruits (*Prunus domestica* L.) measuring 3.5–4.0 cm in diameter, harvested at 90% maturity, and free from mechanical damage or mildew, were harvested from Kashgar Region, Xinjiang. The fruits were stored in the cold storage facility of the College of Food Science and Pharmaceutical Sciences at Xinjiang Agricultural University. All chemical reagents and solvents used were of analytical grade and purchased from Beijing Solarbio Science & Technology Co., Ltd. (Beijing, China).

#### 2.1.2. Experimental Equipment

The TA. XT plus texture analyzer was provided by Microstable Instruments Co., Ltd. (Beijing, China). The PHB-5 Digital pH Meter was purchased from Qiwei Instruments Co., Ltd. (Hangzhou, China). A UV-7504 Ultraviolet-Visible Spectrophotometer was obtained from Shanghai Precision Scientific Instruments Co., Ltd. (Shanghai, China). The BMO5 Handheld Refractometer was sourced from Shangtiance Measurement and Testing Co., Ltd. (Shenzhen, China). The FlavourSpec^®^ GC-IMS system was manufactured by G.A.S. (Gesellschaft für Analytische Sensorsysteme mbH, Dortmund, Germany). The H1850-R High-Speed Benchtop Refrigerated Centrifuge was supplied by Xiangyi Laboratory Instrument Development Co., Ltd. (Changsha, China). A BSA224S Electronic Analytical Balance was procured from Sartorius Scientific Instruments Co., Ltd. (Beijing, China). The SP902S Homogenizer for cell wall disruption was provided by Supor Co., Ltd. (Hangzhou, China). The NR10QC Colorimeter was manufactured by Sanenshi Technology Co., Ltd. (Shenzhen, China).

### 2.2. Sample Preparation

The fresh prune samples were prepared following standardized laboratory procedures: non-decayed fruits were selected, washed, pitted, blanched in boiling water for 3 min to protect color, pulped, and formulated by incorporating 20% xylitol, 0.2% citric acid, and 0.3% low-ester pectin. Fresh prune samples were evenly divided into five experimental groups: the control group (CK) underwent no concentration treatment, while four distinct concentration techniques were applied to obtain processed samples as follows: ATC was conducted using a 1200 W induction cooker under ambient conditions. VTC utilized a rotary evaporator (Model Xiande-2000A, Shanghai Xiande Experimental Instrument Co., Ltd., Shanghai, China) at 60 °C and 0.1 MPa vacuum pressure. VFC employed a vacuum freeze dryer (Model Eco-mini-60, Nanjing Jinshi Co., Ltd., Nanjing, China) with a cold trap temperature of −80 °C and a vacuum level of 13.33 Pa. MVC was performed using a microwave vacuum puffing dryer (Model ORW2.0S-6Z(T), Nanjing Aorun Microwave Technology Co., Ltd., Nanjing, China) at 50 °C and 0.003 MPa vacuum pressure. All concentrated samples were standardized to an equivalent TSS content of approximately 40% by optimizing processing parameters.

### 2.3. Physicochemical Properties Analysis

#### 2.3.1. Determination of Color

The color of jam samples was measured using a colorimeter. The parameters L* (lightness), a* (red–green axis; positive for red, negative for green), and b* (yellow–blue axis; positive for yellow, negative for blue) were determined, while Δ*E* represents the total color difference between French plum jams processed by different concentration methods and the CK, respectively [14]. The Δ*E* was calculated using the following Equation (1):

(1) $$\Delta E=\sqrt{{\left(\Delta L*\right)}^{2}+{\left(\Delta a*\right)}^{2}+{\left(\Delta b*\right)}^{2}}$$

where L*, a*, and b* represent the color-difference parameters of low-sugar prune jam under different concentration methods.

#### 2.3.2. Determination of Browning Intensity (BI) and Soluble Solids (TSS)

The experimental procedure was adapted from the method described by Lee et al. with minor modifications [15]. Specifically, 2 g of jam samples were homogenized with 15 mL of 95% ethanol by vigorous vortexing for 1 min. The mixture was centrifuged at 8000 rpm for 10 min at 4 °C. The supernatant was then carefully decanted and subjected to spectrophotometric analysis, with absorbance measured at 420 nm using a calibrated UV-Vis spectrophotometer (1 cm pathlength quartz cuvette) (Shanghai Precision Scientific Instruments Co., Ltd., Shanghai, China).

The determination of TSS content was performed using a handheld refractometer (Shangtiance Measurement and Testing Co., Ltd., Shenzhen, China).

#### 2.3.3. Determination of Titratable Acid (TA) and L-Ascorbic Acid (AA) Assay

The determination of TA adopted the method described by Li et al. [16], using the acid–base indicator titration method (Method I).

The content of AA was determined using the 2,6-dichlorophenolindophenol (DCPIP) titration method described by Opara et al. [17]. Specifically, 10 g of jam sample was homogenized in a blender and diluted to 100 mL with a 2% oxalic acid solution to stabilize ascorbic acid. The standardized DCPIP solution was then used to titrate the sample until a faint pink color persisted for 30 s without fading. The calculation formula for L-ascorbic acid content is presented in Equation (2):

(2) $$AA=\frac{T\times X\times \left(V-V_{0}\right)}{m}\times 100$$

where the variables are defined as follows: AA = ascorbic acid content in the sample (mg/100 g); V = volume of 2,6-dichlorophenolindophenol solution consumed by the sample (mL); V_0_ = volume consumed by the blank control (mL); T = concentration of 2,6-dichlorophenolindophenol solution (mg/mL); X = dilution factor; m = sample mass (g).

#### 2.3.4. Determination of Dietary Fiber (DF) Assay

DF content was determined using the method described by Ibrahim et al. [18], with minor modifications. Specifically, samples underwent sequential enzymatic digestion using α-amylase, protease, and amyloglucosidase to remove proteins and starch. Ethanol precipitation was then used to separate insoluble dietary fiber from soluble dietary fiber. Both fractions were individually dried and weighed, enabling precise quantification of their respective DF contents via gravimetric analysis.

#### 2.3.5. Determination of Total Phenolic Content (TPC) and Total Flavonoid Content (TFC)

TPC was determined using the Folin–Ciocalteu method described by Esatbeyoglu et al. [19], with minor modifications. A standard calibration curve was constructed using gallic acid solutions at concentrations ranging from 2 to 10 μg/mL. Absorbance was measured at 760 nm, yielding the linear regression equation $y=0.0861x+1.1201(R^{2}=0.9984)$. Results were expressed as milligrams of gallic acid equivalents per 100 g of jam sample (mg/100 g).

TFC was analyzed using a colorimetric method adapted from Salamatullah et al. [20]. Rutin standard solutions at concentrations of 0.5, 1, 2, 3 μg/mL were used to construct a calibration curve, with absorbance measured at 510 nm. The linear regression equation was $y=0.1186x+0.0082(R^{2}=0.9978)$. TFC results were reported as milligrams of rutin equivalents per 100 g of jam sample (mg/100 g).

### 2.4. Determination of Texture Properties Analysis (TPA)

TPA was performed using a method described by Chen et al. [21] with minor modifications. The TPA parameters were set as follows: pre-test speed of 1.0 mm/s, test speed of 1.0 mm/s, post-test speed of 1.0 mm/s, compression ratio of 50%, trigger force of 5 g, and a P36R cylindrical probe.

### 2.5. Sensory Evaluation

All panelists provided written informed consent prior to participation. Based on the methodology of Zheng et al. [22] with minor modifications, 14 panelists (6 male, 8 female) were selected from the College of Food Science and Pharmacy, Xinjiang Agricultural University, through standardized tests for taste/olfactory acuity and descriptive ability assessments.

Trained panelists used pre-validated sensory descriptors to evaluate the aroma characteristics of prune jam, rating attribute intensity on a 5-point scale (0 = none; 1 = extremely weak/unpleasant; 5 = extremely strong/pleasant). Each sample was evaluated in triplicate by each panelist to minimize variability, with results expressed as mean values per attribute. The final score for each descriptor was calculated as the arithmetic mean of all panelists’ ratings, ensuring consistency across evaluations.

### 2.6. E-Nose Determination

E-nose analysis was performed using an AIRSENSE PEN3 instrument (AIRSENSE Analytics GmbH, Schwerin, Mecklenburg-Vorpommern, Germany) following the method of Zhang et al. [11]. System parameters were set as follows: sampling time per group, 80 s; sensor self-cleaning time, 80 s; sensor zeroing time, 5 s; sample preparation time, 5 s; sample injection flow rate, 300 mL/min; and analysis time, 80 s. Data collected at the end of the 80 s analysis period were used for summarization and analysis. Sensor W1C is sensitive to aromatic components; W5S is highly sensitive to nitrogen oxides; W3C responds to ammonia and aromatic components; W6S is selective for hydrogen; W5C detects alkanes and aromatic components; W1S is sensitive to methane; W1W is responsive to sulfides; W2S detects ethanol; W2W is sensitive to aromatic components and organic sulfides; and W3S responds to alkanes.

### 2.7. E-Tongue Determination

Taste profile analysis was performed using an INSENT SA402B E-tongue system (Insent, Atsugi, Japan) following the method of Bai et al. [23]. The system detected six basic taste attributes: sourness, bitterness, astringency, saltiness, umami, and sweetness. For sample preparation, 40 g of fruit jam was mixed with 120 mL of deionized water and magnetically stirred for 5 min to ensure homogeneity. The mixture was then incubated at room temperature for 30 min to allow flavor compounds to equilibrate, followed by centrifugation at 8000 rpm for 10 min. The supernatant was collected and filtered through a 0.45 μm membrane before analysis.

### 2.8. GC-IMS Analysis of Volatile Compounds

Volatile compounds in prune jam samples processed by different concentration methods were analyzed using a FlavourSpec^®^ GC-IMS analyzer (G.A.S. GmbH, Dortmund, Germany) following the method of He et al. [24]. Each sample (2.0 g) was weighed into a 20 mL headspace vial, with triplicate measurements performed to ensure reproducibility. Samples were thermally equilibrated at 50 °C for 15 min in a shaking incubator (200 rpm), followed by automatic injection via a heated syringe (85 °C) in splitless mode. Semi-quantitative analysis utilized 2-methyl-3-heptanone as an internal standard, with 20 μL of a 10 ppm ethanolic solution added to each vial prior to incubation for calibration.

### 2.9. Statistical Analysis

All experiments were independently repeated at least three times (*n* = 3), with results presented as the mean ± standard deviation. Statistical analyses, including ANOVA, Duncan’s multiple range test (*p* = 0.05), principal component analysis (PCA), and partial least squares discriminant analysis (PLS-DA), were conducted using IBM SPSS Statistics 23. Data visualization was carried out using Origin 2021 software. Additionally, a PCA plot was generated using SIMCA 18 software to visually present the distribution of flavor components among different samples. Prior to PCA, raw data from the E-tongue, the E-nose, and GC-IMS were preprocessed to ensure data comparability. E-tongue and E-nose sensor responses were normalized using Z-score standardization to eliminate scale differences, while GC-IMS volatile compound intensities were baseline-corrected, then normalized to an internal standard, and log-transformed to stabilize variance. For PCA, the variables included 12 electronic tongue taste parameters, 10 electronic nose sensor signals, and GC-IMS volatile compound intensities.

## 3. Results and Discussion

### 3.1. Effects of Different Concentration Methods on Color and Quality of Prune Jam

#### 3.1.1. Changes of CPs

**Figure 1:**
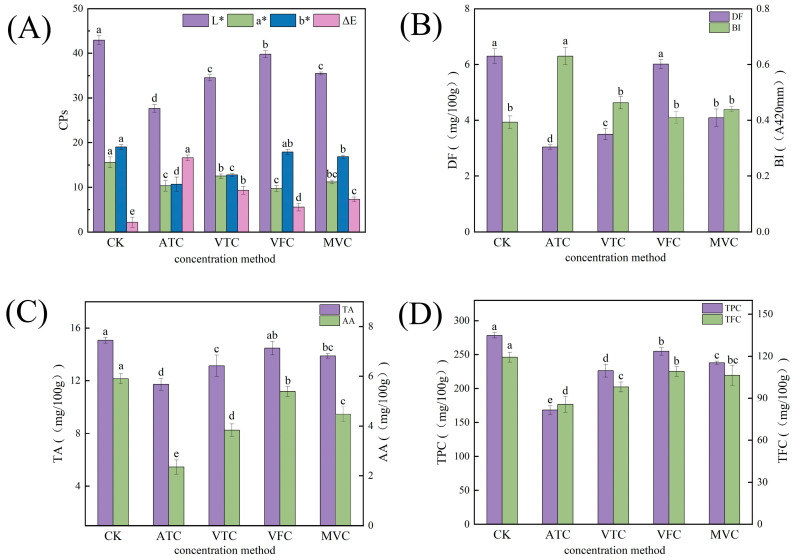
Effects of different concentration methods on (**A**) CPs, (**B**) BI and DF, (**C**) TA and AA, (**D**) TPC and TFC in low-sugar prune jam. The distinct lowercase letters above the bars indicate statistically significant differences (*p* < 0.05) among treatments for each parameter.

CPs serve as crucial indicators for evaluating the visual attributes and consumer acceptability of jams [25]. Changes in the brightness value (L*), red–green value (a*), yellow–blue value (b*), and Δ*E* of prune jam after concentration treatments are shown in Figure 1A. Compared with the CK, the L*, a*, and b* values of samples treated with ATC, VTC, VFC, and MVC decreased significantly (*p* < 0.05). Specifically, L* values were reduced to 64%, 80%, 93%, and 83% of the control; a* values decreased to 66%, 80%, 62%, and 72% of the control; and b* values were 56%, 67%, 94%, and 88% of the control, respectively. Compared with the CK, the ∆E values of the ATC, VTC, VFC, and MVC groups exhibited significant increases of 7.26-fold, 4.28-fold, 2.54-fold, and 3.37-fold, respectively (*p* < 0.05). The ΔE value of the ATC group was 28.5 ± 1.2, which was 7.26 times as high as that of the CK (ΔE = 3.9 ± 0.5), indicating significant browning, and its color was more similar to that of the non-concentrated samples [26]. This is attributed to the decomposition of pigments and the increase in nutritional components during the concentration process, which led to the formation of other pigmented compounds [27,28].

#### 3.1.2. Changes in BI and TSS

BI is the primary factor responsible for the deterioration of color quality in fruit jams [29]. As shown in Figure 1B, the BI of the VTC, VFC, and MVC groups increased significantly (*p* < 0.05) by 1.18-, 1.04-, and 1.12-fold compared with the CK, respectively. In contrast, the ATC group exhibited a 1.6-fold increase in the BI compared to the control. Elevated temperatures accelerate Maillard and caramelization reactions involving reducing sugars, thereby intensifying browning [30]. During ATC processing, continuous heating promotes the degradation of non-reducing sugars (e.g., sucrose) into carbonyl compounds within the jam matrix. These carbonyl compounds react with free amino acids via the Maillard reaction pathway, leading to pronounced browning. In contrast, the non-thermal concentrated VFC group exhibited a 1.04-fold increase in BI. Under low-temperature conditions, reduced biochemical reaction rates limited enzymatic activity, thereby resulting in minimal browning and a color profile closely resembling that of the non-concentrated samples [11].

As shown in Table 1, compared with the CK, treatments ATC, VTC, VFC, and MVC significantly increased the TSS content of prune jam. This is primarily attributed to the continuous concentration process, which induced substantial water evaporation and consequently increased the solute concentration in the samples [31].

#### 3.1.3. Changes in TA and AA Content

TA, a critical indicator of jam quality [26], exhibited significant reductions following concentration treatments (Figure 1C). The TA content in the ATC group decreased to 62.4 mg/100 g, a reduction of 17.6 mg/100 g compared with the CK, and it was only 0.78 times that of the CK. In contrast, the TA content in the VFC group was 76.9 mg/100 g, a decrease of 3.1 mg/100 g compared with the CK, and it was 0.96 times that of the CK. During concentration, elevated temperatures and reduced moisture accelerated the degradation of organic acids, which constitute the TA [32]. In VFC, solute molecules encapsulated in fine ice crystals were expelled during water sublimation under vacuum, minimizing acid volatilization and thermal decomposition. This mechanism effectively preserved TA content by reducing exposure to high temperatures and dehydration stress [33]. This finding is consistent with the research results of Zhang et al. [11] on concentrated sea buckthorn juice. Their study showed that VTC caused the most substantial reduction in TA content. Conversely, non-thermal VFC exhibited significantly superior preservation of TA levels in the concentrated juice.

As a potent antioxidant, AA is highly susceptible to degradation during processing, cooking, and storage [34]. Concentration treatments induced significant AA depletion (Figure 1C): the ATC, VTC, VFC, and MVC groups exhibited 60.1%, 34.9%, 8.6%, and 24.1% reductions in AA content compared to the CK (*p* < 0.05). The AA content in the ATC group decreased to 18.0 mg/100 g, a reduction of 27.2 mg/100 g compared with the CK (45.2 mg/100 g), equivalent to 40% of the CK. In the VFC group, the AA content was 41.2 mg/100 g, a decrease of 4.0 mg/100 g relative to the CK (45.2 mg/100 g), corresponding to 91% of the CK [11]. In a study on pomegranate jam, Velotto et al. [35] demonstrated that freeze-concentrated samples exhibited the highest ascorbic acid retention. In contrast, microwave and vacuum concentration methods led to significant thermal degradation of ascorbic acid, primarily due to exposure to high temperatures. Moreover, ultrasonic treatment was found to potentially accelerate the degradation of ascorbic acid, a pattern consistent with the findings of the present study.

#### 3.1.4. Changes in DF Content

DF, a crucial functional component in food, exhibits diverse physiological benefits [36]. Concentration treatments led to a significant decrease in DF content, as shown in Figure 1B. The DF content in the ATC group decreased to 9.65 mg/100 g (CK: 20.00 mg/100 g), a reduction of 10.35 mg/100 g relative to the CK, equivalent to 48% of the CK. High-temperature conditions in ATC induced thermal and oxidative degradation of DF components, leading to the most pronounced loss. In contrast, the VFC group retained 19.09 mg/100 g of DF (CK: 20.00 mg/100 g), a minor reduction of 0.91 mg/100 g, corresponding to 95% of the CK content. VFC minimized DF degradation via low-temperature vacuum sublimation. For the VTC and MVC groups, partial DF loss occurred under low-temperature conditions and vacuum exposure, with reductions of 8.94 mg/100 g and 13.08 mg/100 g relative to the CK, respectively. The high-temperature environment in ATC induced thermal oxidative degradation and structural breakdown of DF. In contrast, VFC prevented DF cleavage or cross-linkages through low-temperature vacuum sublimation, thereby minimizing DF loss [37].

#### 3.1.5. Changes in TPC and TFC

Phenolic compounds, key bioactive substances in fruits and vegetables, exhibit antioxidant properties [38]. As shown in Figure 1D, the TPC of concentrated prune jam decreased significantly after processing. The TPC in the ATC group decreased to 72.6 mg/100 g (CK: 120.0 mg/100 g), a reduction of 47.4 mg/100 g relative to the CK. In the MVC group, the TPC was 102.8 mg/100 g, corresponding to a 17.2 mg/100 g reduction relative to the CK. The VTC group retained 97.7 mg/100 g of TPC, reflecting a 22.3 mg/100 g decrease relative to the control. Notably, the VFC group maintained a TPC of 110.8 mg/100 g, with only a 9.2 mg/100 g reduction relative to the CK. These results indicate that the concentration temperature is a critical factor in preserving TPC. On one hand, thermal treatment induces degradation or oxidative loss of thermally labile free polyphenols in raw materials, leading to quinone formation. On the other hand, it may release cell wall-bound polyphenols, which undergo subsequent oxidation or aggregation, thereby reducing the TPC [11]. This observation is in accordance with the findings of Velotto et al. [35] on pomegranate jam, wherein freeze concentration was shown to provide superior preservation of the total phenolic content. Conversely, thermal concentration and microwave-assisted processing led to a notable decrease in the retention of phenolic compounds.

Flavonoids, another critical class of antioxidant compounds in fruits and vegetables [39], exhibit a close correlation with TPC. As shown in Figure 1D, compared with the CK, the ATC, VTC, MVC, and VFC treatments led to significant decreases in TFC: 28.25%, 17.80%, 10.85%, and 8.57%, respectively (*p* < 0.05). Meanwhile, VFC achieved the highest retention of TFC. The decline trends of TFC and TPC in prune jam were similar, attributed to the structural vulnerability of flavonoids—their aromatic rings bear multiple phenolic hydroxyl groups, which are prone to thermal oxidation under elevated temperatures. In contrast, the low-temperature vacuum environment in VFC minimizes such oxidation; additionally, the mechanical stress from ice crystal formation during freezing promotes cell wall disruption, releasing bound phenolic acids that partially compensate for TFC loss [40].

### 3.2. Texture Profile Analysis

Texture properties, including hardness, adhesiveness, cohesiveness, gumminess, and chewiness, reflect the sensory quality and structural characteristics of jam [17]. As shown in Table 2, prune jam subjected to concentration treatments exhibited higher hardness compared to the CK. Specifically, the ATC, VTC, MVC, and VFC groups had hardness values of 183.92 ± 5.94 g, 165.58 ± 4.27 g, 158.36 ± 3.89 g, and 122.64 ± 3.15 g, respectively, representing increases of 209%, 143%, 181%, and 70% relative to the control (*p* < 0.05). The ATC group exhibited higher adhesiveness and chewiness than other treatment groups, which can be attributed to moisture evaporation during concentration—this process increased solid content and jam density [41]. In contrast, the VFC group showed the lowest values for all texture parameters (hardness, adhesiveness, cohesiveness, gumminess, chewiness; *p* < 0.05), indicating that VFC-treated jam has enhanced fluidity, facilitating easier spreading in the oral cavity [42].

### 3.3. E-Tongue Analysis

**Figure 2:**
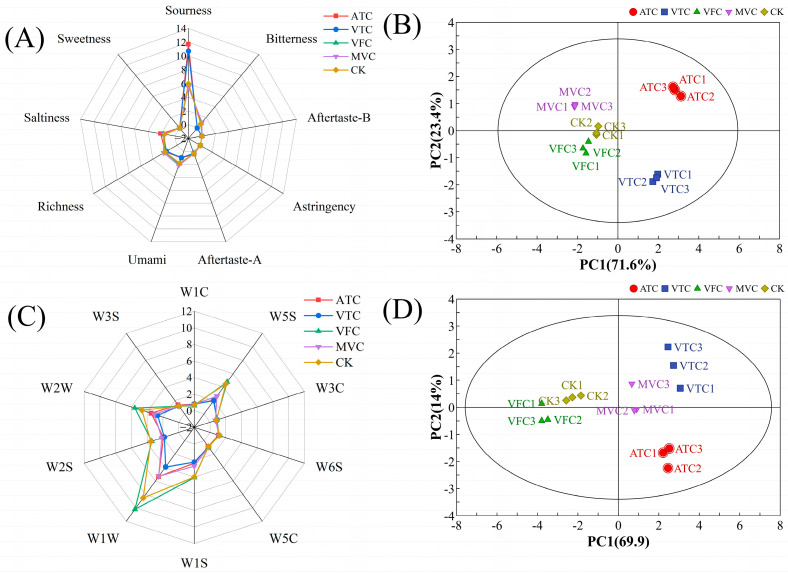
E-tongue radar plot (**A**), E-tongue PCA score plot (**B**), E-nose radar plot (**C**), and E-nose PCA score plot (**D**) for prune jam processed by different concentration methods. In the PCA score plot, the labels 1, 2, and 3 represent three technical replicates of the same treatment group. For example, ATC1–ATC3 denote triplicate samples from the ATC treatment.

The E-tongue, an advanced detection instrument capable of simulating human taste perception [43], indicated that prune jams treated with different concentration methods showed overall similarity in taste indicators (Figure 2A). The E-tongue results showed that the sour sensor response intensity of concentrated jam (12.3 ± 0.5) was significantly higher than that of the control group (5.8 ± 0.3), which indicated an increase in organic acid content. The umami sensor response intensity (8.7 ± 0.4) was the second highest among all sensors, suggesting the retention or formation of amino acids. These results indicated that after concentration, the jams had enhanced sourness, umami, and saltiness. The ATC group exhibited the highest sourness value (approximately 12), followed by VTC (around 10), while VFC (5.99) was most similar to the control group (CK). This suggests that ATC treatment intensified sourness, whereas VFC preserved the original flavor profile most effectively, likely due to minimal thermal disruption of volatile organic acids and key flavor compounds.

Regarding the PCA, Figure 2B reveals a cumulative variance explained of 92.9% (PC1: 71.6%, PC2: 21.3%, *p* < 0.001), indicating that PC1 and PC2 captured nearly all sample variability. Samples clustered distinctly along the principal components, demonstrating significant differences in taste composition that reflected the overall taste profiles of jams under different concentration treatments. The clustering pattern in the PCA score plot (Figure 2B) showed that ATC and VTC samples formed a distinct cluster, while MVC, VFC, and CK samples grouped into another distinct cluster, indicating high intra-cluster flavor similarity. This finding validated the E-tongue’s capability to discriminate taste profiles of prune jams under different concentration treatments [36].

### 3.4. E-Nose Analysis

The E-nose, an analytical technique mimicking human olfaction [44], addresses limitations of sensory variability and the complexity inherent in chromatographic methods. Notable variations in sensor response values were observed for jam samples after concentration treatment (Figure 2C). Among the 10 sensors, the sulfides (W1W), aromatic components (W2W), nitrogen oxides (W5S), and methane (W1S) sensors exhibited the most pronounced responses, with the sulfides sensor (W1W) showing the highest response intensity. Furthermore, the five treated sample groups displayed comparable response values for the aromatic components (W1C), ammonia (W3C), hydrogen (W6S), and alkanes (W5C, W3S) sensors when compared to the CK. The study identified the primary volatile odor compounds in prune jam after concentration treatment as inorganic sulfides, aromatic components (organic sulfides), nitrogen oxides, alcohols, and methyl compounds. The corresponding sensor response intensities served as critical indicators for differentiating post-concentration jam samples based on their unique volatile profiles [41].

PCA of the E-nose dataset (Figure 2D) revealed that PC1 explained 69.9% of the variance, while PC2 accounted for 14%, yielding a cumulative variance of 83.9%. This high cumulative variance indicates that the first two principal components effectively captured the majority of information from the original sensor response data, highlighting PC1 as the primary driver of variation in volatile profiles among jam samples. The results demonstrated significant differences in sensor response intensities across groups, indicating that the E-nose system could effectively discriminate overall odor profiles of jam samples after concentration treatment—even with limitations in identifying specific volatile compounds [45]. To address this specificity, subsequent studies will utilize advanced analytical techniques such as GC-IMS for in-depth characterization of volatile components.

### 3.5. Analysis of Volatile Component Changes in Prune Jam After Concentration Treatment Based on GC-IMS

**Figure 3:**
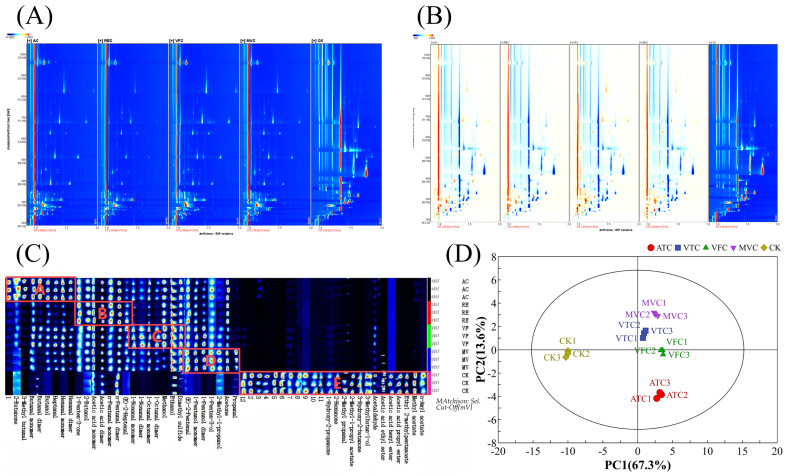
Two-dimensional GC-IMS plot (**A**), GC-IMS difference spectrum (**B**), volatile component fingerprint (**C**), and PCA score plot of volatile compounds (**D**) for prune jam processed by different concentration methods. In the volatile component fingerprint, the substances in Region A, Region B, Region C, Region D, and Region E have the highest concentrations in ATC, VTC, VFC, MVC, and CK respectively. In the PCA score plot, the labels 1, 2, and 3 represent three technical replicates of the same treatment group. For example, ATC1–ATC3 denote triplicate samples from the ATC treatment.

The volatile component profiles of prune jam under different concentration treatments were analyzed using GC-IMS (Figure 3). In the GC-IMS two-dimensional spectrum (Figure 3A), the vertical axis represents GC retention time, while the horizontal axis indicates ion migration time. The GC-IMS spectrum background is blue, with a red vertical line at a horizontal coordinate of 1.0 denoting the normalized reactive ion peak (RIP) for data calibration [22]. ATC and VTC samples exhibited similarity in spot intensity and distribution, whereas VFC and MVC samples formed distinct clusters, highlighting clear differences from the ATC/VTC group. When the CK was set as the reference (Figure 3B), inter-sample variations became more pronounced. The background colors—white, blue, and red—represent volatile compound concentrations equivalent to, lower than, and higher than those of the reference CK, respectively. Darker spot colors correspond to higher peak intensities of the respective volatile compounds, indicating greater abundance or sensitivity in detection [24]. Compared with the CK, ATC, VTC, VFC, and MVC samples exhibited a higher proportion of blue regions in the GC-IMS spectra, indicating lower volatile compound concentrations under most measurement conditions and drift time ranges. Variations in peak positions, intensities, and color distributions—excluding the normalized reactive ion peak (red vertical line)—suggested both common and unique volatile components across samples, with differential abundance levels [45].

**Figure 4:**
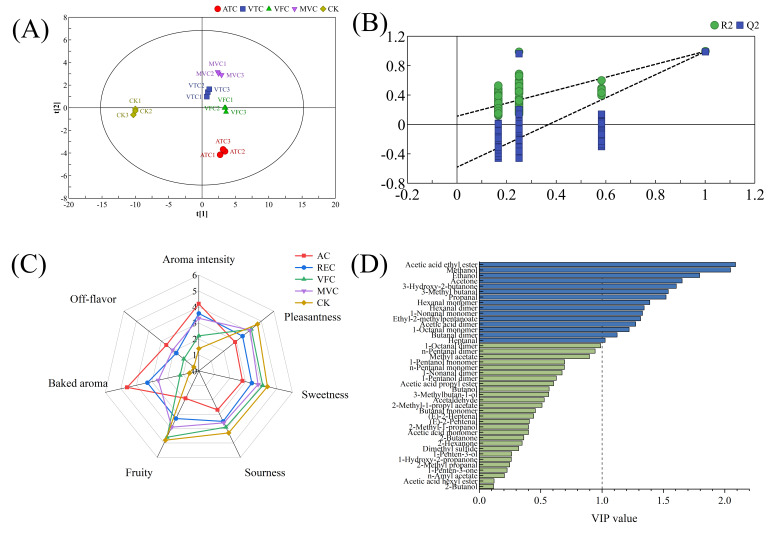
PLS-DA score plot (*R*^2^ = 0.993, *Q*^2^ = 0.985) (**A**); permutation test results for model validation (200 iterations, intercept *R*^2^ = 0.112, intercept *Q*^2^ = −0.581) (**B**); sensory evaluation radar plot of l prune jam under different concentration methods (**C**); variable importance in projection (VIP) score plot (**D**). In the PCA score plot, the labels 1, 2, and 3 represent three technical replicates of the same treatment group. For example, ATC1–ATC3 denote triplicate samples from the ATC treatment.

**Figure 5:**
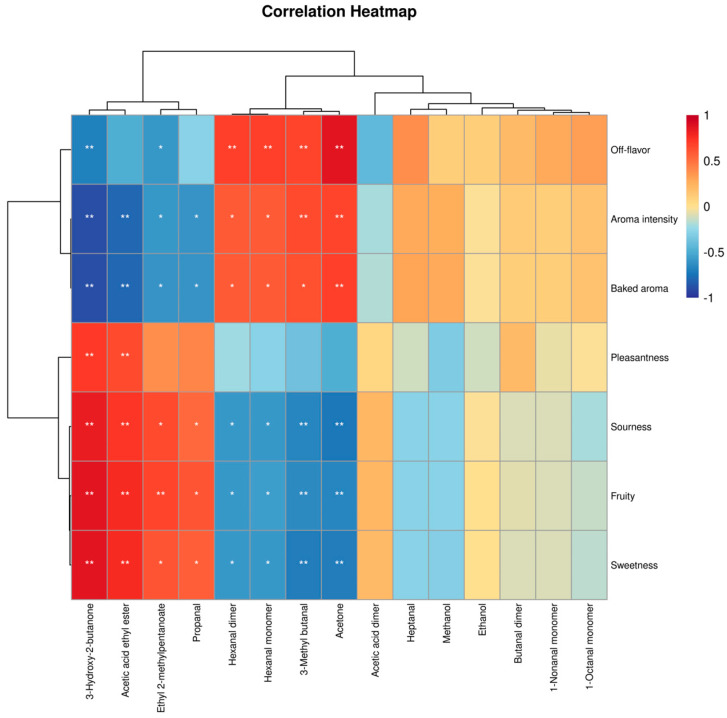
Heatmap of Spearman correlations between sensory attributes and volatile compounds in prune jam under different concentration methods. Correlation heatmap. ‘*’ and ‘**’ denote significant correlations at the (*p* < 0.05) and (*p* < 0.01) levels, respectively.

Further analysis of the volatile compound fingerprint (Figure 3C) revealed that each row in the profile represented all selected signal peaks for an individual sample, while each column corresponded to the same volatile compound across different samples. Brighter colors indicated higher concentrations, and darker colors denoted lower concentrations [43]. A total of 42 volatile compounds were identified across the five samples. Non-thermal treatments (VFC, MVC) preserved higher levels of fruit-associated esters (e.g., ethyl acetate), whereas thermal treatments (ATC, VTC) increased aldehyde and alcohol derivatives associated with off-flavor characteristics. This distinction underscores the influence of processing methods on volatile profile preservation and degradation pathways. As listed in Table 3, these compounds were classified into six chemical families based on structural and functional properties: 17 aldehydes, 9 alcohols, 7 esters, 6 ketones, 2 organic acids, and 1 sulfur-containing compound. Aldehydes and alcohols comprised the largest subclasses, accounting for the majority of identified volatiles. High-abundance volatile compounds in each sample were localized to distinct regions (Regions A–D corresponding to ATC, VTC, VFC, and MVC, respectively) in the GC-IMS contour plots. Region E, containing compounds with the highest abundances in the CK sample, included ethyl acetate, 3-hydroxy-2-butanone, ethyl 2-methylpentanoate, and 3-methylbutan-1-ol. As detailed in Table 3, the CK sample exhibited the highest ester content, with ethyl acetate (196.05 ± 10.52 μg/kg) being the most abundant—significantly surpassing all treatment groups (*p* < 0.05). Esters, renowned for their sweet and fruity aromatic profiles, serve as key contributors to the characteristic flavor of fruit-based products [11]. Following concentration treatments, ester concentrations decreased significantly (*p* < 0.05), whereas aldehyde and alcohol abundances increased notably (*p* < 0.05). VFC and ATC samples exhibited the highest relative aldehyde levels, presumably due to hydrogen abstraction and alcohol oxidation during water removal—processes that likely promote aldehyde formation and contribute to flavor modifications in prune jam. A PLS-DA model was constructed using GC-IMS data to investigate the correlation between volatile compounds and sensory attributes of low-sugar prune jam. The PLS-DA model effectively discriminated among the four jam products (Figure 4A,B), exhibiting strong variance explanatory power and cross-validation predictive ability (*R*² = 0.993, *Q*² = 0.985). QDA) was conducted to characterize the aroma profiles, identifying seven critical sensory attributes: aroma intensity, pleasantness, sweetness, sourness, fruitiness, baked aroma, and off-flavor (Figure 4C). The ATC sample displayed dominant baked aroma, off-flavor, and aroma intensity but weaker fruitiness, pleasantness, and sweetness compared to other groups.Spearman correlation analysis, visualized as a heatmap (Figure 5), further elucidated the relationships between 15 VIP-selected volatile compounds and sensory attributes. Compounds such as acetone, ethyl acetate, and propanal exhibited positive correlations with desirable sensory traits like fruitiness and pleasantness. Specifically, acetone, hexanal monomer, hexanal dimer, and 3-methylbutanal—key thermal degradation byproducts—exhibited strong positive correlations (*p* < 0.05) with off-flavor, elevated aroma intensity, and baked notes.

**Table 3:** Composition and content of volatile flavor compounds in prune jam under different concentration treatments, and 15 key volatile compounds screened by sensory-group correlation and variable importance in projection (VIP > 1).

No	Compound	VIP	Content (ug/kg)					Odor Description
			ATC	VTC	VFC	MVC	CK	
1	ATCetic acid ethyl ester	2.0876	6.14 ± 0.23 ^c^	5.3 ± 0.28 ^c^	8.47 ± 0.46 ^c^	32.24 ± 3.15 ^b^	196.05 ± 10.52 ^a^	fresh, fruity, sweet, grassy
2	Methanol	2.04638	10.93 ± 0.43 ^c^	12.37 ± 0.15 ^b^	17.14 ± 0.85 ^a^	4.06 ± 0.96 ^d^	0.31 ± 0.03 ^e^	alcohol, pungent
3	Ethanol	1.79529	28.57 ± 0.49 ^d^	31.29 ± 0.42 ^c^	48.31 ± 0.23 ^a^	40.51 ± 0.84 ^b^	20.44 ± 1.03 ^e^	aromaticity
4	Acetone	1.65265	15.57 ± 0.94 ^b^	14.77 ± 0.24 ^b^	8.99 ± 0.13 ^c^	17.56 ± 1.98 ^a^	1.18 ± 0.11 ^d^	fresh, apple, pear
5	3-Hydroxy-2-butanone	1.60444	2.88 ± 0.10 ^b^	2.36 ± 0.11 ^b^	4.23 ± 0.20 ^b^	3.53 ± 0.31 ^b^	132.86 ± 15.47 ^a^	butter, cream
6	3-Methyl butanal	1.53947	4.75 ± 0.17 ^a^	0.53 ± 0.01 ^b^	0.58 ± 0.03 ^b^	0.53 ± 0.12 ^b^	0.26 ± 0.01 ^c^	chocolate, fat
7	Propanal	1.52113	0.59 ± 0.19 ^b^	0.52 ± 0.08 ^b^	0.84 ± 0.07 ^b^	5.13 ± 1.26 ^a^	1.27 ± 0.03 ^b^	pungent, green grassy
8	Hexanal monomer	1.38859	9.35 ± 0.23 ^a^	3.42 ± 0.15 ^d^	8.52 ± 0.31 ^b^	6.36 ± 0.29 ^c^	1.31 ± 0.07 ^e^	fresh, green, fat, fruity
9	Hexanal dimer	1.34278	5.27 ± 0.09 ^a^	0.65 ± 0.08 ^d^	3.75 ± 0.54 b	1.8 ± 0.13 ^c^	0.4 ± 0.01 ^d^	fresh, green, fat, fruity
10	1-Nonanal monomer	1.34278	8.55 ± 0.90 ^c^	4.44 ± 0.62 ^d^	14.30 ± 0.69 ^a^	9.97 ± 0.24 ^b^	1.46 ± 0.08 ^e^	rose, citrus, strong oily
11	Ethyl 2-methylpentanoate	1.31415	1.72 ± 0.17 ^b^	1.89 ± 0.20 ^b^	1.85 ± 0.14 ^b^	1.98± 0.25 ^b^	89.93 ± 1.31 ^a^	fresh fruit flavor, cucumber, apple peel, pineapple
12	Acetic acid dimer	1.274	18.7 ± 1.23 ^b^	18.8 ± 0.52 ^b^	18.08 ± 0.88 ^b^	15.68 ± 0.61 ^b^	24.59 ± 5.14 ^a^	spicy
13	1-Octanal monomer	1.22022	9.79 ± 0.48 ^b^	5.28 ± 0.43 ^c^	13.71 ± 0.05 ^a^	10.04 ± 0.53 ^b^	2.06 ± 0.06 ^d^	aldehyde, waxy, citrus, orange, fruity, fatty
14	Butanal dimer	1.12242	3.37 ± 0.10 ^a^	0.65 ± 0.03 ^e^	2.37 ± 0.12 ^b^	0.91 ± 0.02 ^d^	1.59 ± 0.07 ^c^	pungent, fruity, green leaf
15	Heptanal	1.02522	4.04 ± 0.16 ^b^	0.93 ± 0.06 ^d^	4.31 ± 0.18 ^a^	2.52 ± 0.19 ^c^	0.51 ± 0.05 ^e^	fresh, aldehyde, fatty, green herbs, wine, fruity
16	Acetaldehyde		1.4 ± 0.10 ^c^	0.82 ± 0.01 ^c^	2.44 ± 0.04 ^b^	1.59 ± 0.09 ^bc^	3.78 ± 1.05 ^a^	green, slight fruity
17	2-Butanone		0.48 ± 0.02 ^a^	0.21 ± 0.00 ^c^	0.3 ± 0.02 ^b^	0.15 ± 0.01 ^d^	0.11 ± 0.02 ^e^	fruity, camphor
18	1-Penten-3-ol		0.55 ± 0.05 ^b^	0.38 ± 0.05 ^c^	0.53 ± 0.03 ^b^	0.63 ± 0.04 ^a^	0.11 ± 0.02 ^d^	ethereal, green, tropical fruity
19	3-Methylbutan-1-ol		0.83 ± 0.07 ^b^	0.92 ± 0.06 ^b^	0.91 ± 0.09 ^b^	0.96 ± 0.03 ^b^	17.35 ± 0.93 ^a^	whiskey, banana, fruity
20	1-Pentanol monomer		3.69 ± 0.29 ^b^	3.88 ± 0.18 ^b^	6.02 ± 0.10 ^a^	6.28 ± 0.13 ^a^	1.05 ± 0.22 ^c^	balsamic
21	1-Pentanol dimer		0.80 ± 0.09 ^b^	0.78 ± 0.04 ^b^	2.06 ± 0.08 ^a^	2.02 ± 0.01 ^a^	0.37 ± 0.02 ^c^	balsamic
22	Acetic acid monomer		16.64 ± 0.17 ^c^	18.01 ± 0.26 ^b^	18.92 ± 0.18 ^a^	18.53 ± 0.35 ^ab^	15.85 ± 0.83 ^c^	spicy
23	2-Methyl-1-propanol		0.18 ± 0.02 ^c^	0.16 ± 0.02 ^c^	0.42 ± 0.03 ^b^	0.59 ± 0.02 ^a^	0.19 ± 0.01 ^c^	fresh, alcoholic, leather
24	Butanol		2.29 ± 0.04 ^a^	1.27 ± 0.09 ^d^	1.86 ± 0.05 ^b^	1.59 ± 0.04 ^c^	0.39 ± 0.04 ^e^	wine
25	Methyl acetate		3.51 ± 0.09 ^b^	0.92 ± 0.04 ^c^	1.76 ± 0.03 ^c^	1.92 ± 0.49 ^c^	36.8 ± 1.36 ^a^	ester, green
26	Acetic acid propyl ester		0.36 ± 0.04 ^b^	0.43 ± 0.04 ^b^	0.69 ± 0.11 ^b^	0.52 ± 0.03 ^b^	18.68 ± 0.54 ^a^	fruity, pear
27	2-Butanol		0.76 ± 0.06 ^b^	0.85 ± 0.06 ^a^	0.82 ± 0.03 ^a^	0.86 ± 0.06 ^a^	0.55 ± 0.24 ^b^	fruity
28	2-Methyl-1-propyl acetate		0.39 ± 0.05 ^b^	0.36 ± 0.01 ^b^	0.41 ± 0.03 ^b^	0.39 ± 0.03 ^b^	13.6 ± 0.65 ^a^	fruity, raw pear and raspberry
29	1-Octanal dimer		2.39 ± 0.23 ^b^	0.81 ± 0.08 ^c^	5.21 ± 0.29 ^a^	2.55 ± 0.19 ^b^	0.56 ± 0.15 ^c^	aldehyde, waxy, citrus, orange, fruity, fatty
30	Acetic acid hexyl ester		0.2 ± 0.03 ^b^	0.24 ± 0.02 ^b^	0.24 ± 0.02 ^b^	0.23 ± 0.01 ^b^	0.94 ± 0.08 ^a^	fruity, green, apple, banana, sweet
31	2-Hexanone		0.76 ± 0.06 ^b^	0.5 ± 0.05 ^c^	0.7 ± 0.06 ^bc^	0.64 ± 0.07 ^bc^	6.29 ± 0.24 ^a^	fruity, fungal, meaty, buttery
32	Dimethyl sulfide		4.26 ± 0.25 ^b^	4.03 ± 0.06 ^b^	5.15 ± 0.10 ^a^	4.95 ± 0.23 ^a^	1.98 ± 0.63 ^c^	cabbage, sulfur, gasoline
33	Butanal monomer		2.12 ± 0.08 ^a^	1.28 ± 0.03 ^c^	2.25 ± 0.02 ^a^	1.94 ± 0.14 ^b^	0.34 ± 0.07 ^d^	pungent, fruity, green leaf
34	2-Methyl propanal		0.38 ± 0.11 ^b^	0.14 ± 0.01 ^b^	0.44 ± 0.08 ^b^	0.24 ± 0.02 ^b^	2.57 ± 1.32 ^a^	banana, melon, slightly nutty
35	n-Pentanal monomer		4.76 ± 0.06 ^b^	5.74 ± 0.12 ^a^	3.63 ± 0.09 ^c^	4.59 ± 0.14 ^b^	0.81 ± 0.02 ^d^	green grassy, faint banana, pungent
36	n-Pentanal dimer		11.32 ± 0.41 ^c^	12.54 ± 0.27 ^b^	10.27 ± 0.42 ^d^	10.35 ± 0.34 ^d^	17.78 ± 0.38 ^a^	green grassy, faint banana, pungent
37	1-Penten-3-one		1.43 ± 0.08 ^c^	1.68 ± 0.09 ^a^	1.52 ± 0.05 ^bc^	1.62 ± 0.13 ^ab^	0.12 ± 0.01 ^d^	strong pungent odors
38	n-Amyl acetate		0.14 ± 0.03 ^b^	0.15 ± 0.02 ^b^	0.19 ± 0.00 ^b^	0.15 ± 0.02 ^b^	2.22 ± 0.39 ^a^	bananas, apples, pears
39	(E)-2-Pentenal		1.47 ± 0.12 ^b^	1.82 ± 0.17 ^a^	1.37 ± 0.03 ^b^	2.12 ± 0.32 ^a^	0.42 ± 0.02 ^c^	potato, peas
40	1-Nonanal dimer		0.98 ± 0.17 ^b^	0.47 ± 0.01 ^c^	2.37 ± 0.24 ^a^	1.08 ± 0.09 ^b^	0.38 ± 0.03 ^c^	rose, citrus, strong oily
41	(E)-2-Heptenal		0.63 ± 0.10 ^b^	1.13 ± 0.20 ^a^	1.45 ± 0.37 ^a^	1.30 ± 0.06 ^a^	0.50 ± 0.05 ^b^	spicy, green vegetables, fresh, fatty
42	1-Hydroxy-2 propanone		0.57 ± 0.09 ^bc^	0.42 ± 0.04 ^c^	0.85 ± 0.02 ^b^	0.66 ± 0.02 ^bc^	2.86 ± 0.41 ^a^	pungent, caramel, fresh

Note: Values are presented as mean ± S.D. Odor descriptions are derived from the ChemicalBook database. M = monomer; D = dimer; RI = retention index; Rt = retention time. Different lowercase letters in the same column indicate significant differences (*p* < 0.05).

Based on variations in volatile profiles, samples were classified into five distinct groups—CK, VFC, VTC, ATC, and MVC—in the hierarchical clustering analysis (Figure 3D). In the PCA score plot, the CK sample was clearly separated from the four concentrated treatment groups, indicating that concentration treatments significantly influenced aroma-related volatile compounds. The principal components, PC1 and PC2, accounted for 67.3% and 13.6% of the variance, respectively, with a cumulative contribution rate of 80.9%. These components represent the primary aroma characteristics of all samples. In the PCA plot, the relatively close proximity of VTC and VFC sample points implies potential disparities in the quantity or nature of specific volatile compounds. Although these samples are located in the lower regions of the plot, they still maintain inter-sample similarity, as previously reported [46]. By contrast, MVC, which is characterized by its unique concentration method, displayed distinct volatile compound profiles compared to other methods. It occupied a relatively upper position in the PCA plot, and the clustering of MVC samples indicated a stable pattern of volatile compound features associated with this technique. ATC samples were situated in the lower part of the plot, spatially segregated from other groups. This spatial distribution likely reflects the alterations in volatile compounds under high-temperature conditions, including the loss of desirable flavor substances and the generation of unwanted volatile components. Collectively, these findings strongly indicate significant differences in volatile composition between the ATC group and the others. These PCA results corroborated the findings from the volatile fingerprint analysis. Thermal concentration under acidic conditions promoted the degradation of esters and alcohols, generating odor-active acidic compounds associated with off-flavors. In contrast, non-thermal concentration methods better maintained the original prune flavor profile by minimizing thermal stress-induced chemical alterations, as evidenced by higher retention of characteristic esters and lower levels of degradation byproducts [47]. When analyzing the impacts of various concentration techniques on volatile components, thermal concentration was determined to significantly reduce ester content while concurrently increasing the levels of aldehydes and alcohols. This outcome is congruent with the research findings of Zhang et al. [11] on concentrated sea buckthorn juice, in which thermal processing detrimentally affected flavor compounds, whereas non-thermal methods were more effective in preserving the initial flavor profile. Significantly, the present study extends this body of knowledge by utilizing PCA to systematically elucidate the unique volatile compound profiles associated with each concentration method in fruit jam processing. This methodological approach offers novel mechanistic perspectives on the flavor transformation processes that occur during the concentration stage.

## 4. Conclusions

This study systematically investigates the effects of four concentration methods—ATC, VTC, VFC, and MVC—on the physicochemical quality parameters and key volatile compounds of prune jam. For the first time, an integrated analytical framework was established by combining the E-tongue, the E-nose, and GC-IMS with multivariate statistical analysis methods, such as PCA, PLS-DA, and VIP. The results demonstrate substantial degradation of key nutrients—AA, TA, DF, TPC, and TFC—under thermal concentration methods (ATC, VTC). ATC induced a drastic 60.1% reduction in AA content and 51.75% loss of DF, whereas non-thermal methods (notably VFC) exhibited superior nutrient retention, preserving 91.4% of AA and limiting DF loss to 4.55%. The retention efficiency of phenolic compounds by VFC was 2.3–4.7-fold higher than that by ATC (Figure 1B–D). Thermal processing triggered intense non-enzymatic browning, with ATC-treated samples showing a 160% increase in BI and a 7.26-fold higher ΔE compared to the CK. The ΔE value of the VFC group was 5.87, which was 2.54 times as high as that of the CK (ΔE = 2.31), indicating its effective inhibition of color degradation via the low-temperature sublimation mechanism. The chromatic characteristics of VFC-treated samples closely matched those of unconcentrated products (Figure 1A,B). GC-IMS volatile fingerprinting identified 42 compounds, revealing that thermal methods caused 86–97% degradation of esters (e.g., ethyl acetate) but 2.1–5.8-fold accumulation of aldehydes (e.g., hexanal). VFC retained 91.5% of characteristic esters (ethyl acetate content: 13.4-fold higher than ATC) and suppressed off-flavor aldehydes/alcohols (Figure 3C, Table 3). Through VIP analysis, 15 key flavor compounds (e.g., ethyl acetate, methanol) were identified. Non-thermal concentration methods exhibited 38–62% lower VIP scores for off-flavor compounds compared to thermal methods, consistent with sensory evaluations that documented enhanced fruity aroma pleasantness and reduced bitterness (Figure 4C,D). Compared to thermal methods, VFC demonstrated significantly superior performance in retaining bioactive components, inhibiting non-enzymatic browning, and preserving characteristic fruity aromas. These findings provide a scientific foundation for sustainable processing of prune jam. By addressing the research gap in systematic evaluation of non-thermal technologies for prune processing, this study also highlights that the multidimensional analytical framework is applicable to optimizing production processes for other thermally sensitive fruit products.

## Figures and Tables

**Table 1:** Impact of different concentration techniques on TSS of prune jam.

Sample	CK	ATC	VTC	VFC	MVC
TSS/%	9.34 ± 0.45 ^b^	44.78 ± 0.36 ^a^	43.76 ± 0.22 ^a^	41.91 ± 0.11 ^a^	42.77 ± 0.37 ^a^

Note: Data are shown as mean ± standard deviation (S.D.). In the same row, different letters denote significant differences (*p* < 0.05) determined by one-way analysis of variance (ANOVA) followed by Tukey’s honestly significant difference test.

**Table 2:** Impact of different concentration techniques on textural attributes of prune jam.

Sample	Hardness/g	Adhesiveness/(g.s)	Cohesiveness/g	Gumminess/g	Chewiness/g
CK	59.53 ± 3.24 ^e^	−8.60 ± 0.45 ^a^	0.68 ± 0.02 ^c^	49.60 ± 1.85 ^d^	51.61 ± 4.32 ^e^
ATC	183.92 ± 5.94 ^a^	−155.89 ± 2.73 ^e^	0.96 ± 0.05 ^a^	183.45 ± 5.53 ^a^	190.17 ± 5.13 ^a^
VTC	144.47 ± 5.09 ^c^	−112.57 ± 2.44 ^c^	0.85 ± 0.03 ^b^	153.93 ± 12.80 ^b^	136.94 ± 2.07 ^c^
VFC	100.91 ± 2.03 ^d^	−62.20 ± 5.17 ^b^	0.73 ± 0.03 ^c^	120.78 ± 4.10 ^c^	97.73 ± 5.31 ^d^
MVC	167.40 ± 1.49 ^b^	−140.76 ± 8.83 ^d^	0.87 ± 0.01 ^b^	161.04 ± 7.23 ^b^	162.03 ± 5.61 ^b^

Note: Data are presented as mean ± SD. In the same column, different letters denote significant differences (*p* < 0.05) determined by one-way analysis of variance (ANOVA) followed by Tukey’s HSD test. A negative adhesiveness value indicates that the sample demonstrates adhesion resistance to the probe.

## Data Availability

The original contributions presented in the study are included in the article, further inquiries can be directed to the corresponding author.

## Abbreviations

The following abbreviations are used in this manuscript:

ATC	atmospheric thermal concentration
VFC	vacuum freezing concentration
MVC	microwave vacuum concentration
VTC	vacuum thermal concentration
CK	control group
GC-IMS	gas chromatography–ion mobility spectrometry
PCA	principal component analysis
BI	browning intensity
CPs	color parameters
TA	titratable acidity
AA	ascorbic acid
DF	dietary fiber
TPC	total phenolic content
TFC	total flavonoid content
TPA	texture properties analysis
PLS-DA	partial least squares discriminant analysis
VIP	variable importance in projection
QDA	quantitative descriptive analysis
